# Fear of COVID-19 among homeless individuals in Germany in mid-2021

**DOI:** 10.3389/fpubh.2022.915965

**Published:** 2022-08-10

**Authors:** André Hajek, Franziska Bertram, Victoria van Rüth, Katharina Dost, Wiebke Graf, Anna Brenneke, Veronika Kowalski, Klaus Püschel, Christine Schüler, Benjamin Ondruschka, Fabian Heinrich, Hans-Helmut König

**Affiliations:** ^1^Department of Health Economics and Health Services Research, University Medical Center Hamburg-Eppendorf, Hamburg, Germany; ^2^Department of Legal Medicine, University Medical Center Hamburg-Eppendorf, Hamburg, Germany; ^3^Deutsches Rotes Kreuz Kreisverband Hamburg Altona und Mitte e.V, Hamburg, Germany

**Keywords:** homeless, fear of COVID-19, COVID-19 worry, anxiety of COVID-19, coronavirus anxiety, SARS-CoV-2

## Abstract

**Aims:**

To investigate the prevalence and the correlates of fear of COVID-19 among homeless individuals.

**Methods:**

We used data from the “national survey on psychiatric and somatic health of homeless individuals during the COVID-19 pandemic” (NAPSHI-study) which took place in several large cities in Germany in Mid-2021 (*n* = 666 in the analytical sample). Mean age equaled 43.3 years (SD: 12.1 years), ranging from 18 to 80 years. Multiple linear regressions were performed.

**Results:**

In our study, 70.9% of the homeless individuals reported no fear of COVID-19. Furthermore, 14.0% reported a little fear of COVID-19, 8.4% reported some fear of COVID-19 and 6.7% reported severe fear of COVID-19. Multiple linear regressions revealed that fear of COVID-19 was higher among individuals aged 50–64 years (compared to individuals aged 18–29 years: *β* = 0.28, *p* < 0.05), among individuals with a higher perceived own risk of contracting the coronavirus 1 day (*β* = 0.28, *p* < 0.001) as well as among individuals with a higher agreement that a diagnosis of the coronavirus would ruin his/her life (*β* = 0.15, *p* < 0.001).

**Conclusions:**

Only a small proportion of homeless individuals reported fear of COVID-19 in mid-2021 in Germany. Such knowledge about the correlates of higher levels of fear of COVID-19 may be helpful for addressing certain risk groups (e.g., homeless individuals aged 50–64 years). In a further step, avoiding extraordinarily high levels of fear of COVID-19 may be beneficial to avoid irrational thinking and acting regarding COVID-19 in this group.

## Introduction

During the year 2020, 256,000 individuals were homeless in Germany (excluding refugees) (1). It has been revealed that homeless individuals have higher prevalence rates of, for example, infectious diseases or mental illnesses when compared to the general adult population (2, 3). Additionally, they often die earlier (4). In light of the fact that the number of homeless individuals rose in the preceding years, the relevance of this group should be emphasized.

Previous research has assumed that homeless individuals may reflect “super spreaders” in times of the COVID-19 pandemic—given the fact that they are quite mobile (5). Moreover, factors such as reactance toward hospital treatment and potential difficult isolation or quarantine for homeless individuals may highlight the general relevance of this group in times of the pandemic (6).

Thus far, there is very limited knowledge regarding the fear of COVID-19 among homeless individuals. In fact, we are only aware of one single study examining fear of COVID-19 among homeless individuals (7). More precisely, this previous study (7) showed that more than one-half of homeless individuals living in Hamburg (second largest city in Germany) reported no fear of COVID-19 in May 2020. However, this study referred to an early stage of the pandemic and is limited to one city in Germany. In this current study, we aimed to examine the fear of COVID-19 among homeless individuals in several large German cities in mid-2021 (i.e., a later stage of the pandemic).

With regard to relevance, such knowledge about fear of COVID-19 among homeless individuals is of great importance since high levels of fear of COVID-19 can contribute to suicide (8). Moreover, fear of COVID-19 can contribute to death anxiety (9)—at the same time, low levels of fear of COVID-19 may result in inconsiderate or careless behavior which could lead to super-spreading. Such knowledge may therefore be of importance for policymakers, social services, physicians, and public health practitioners. More precisely, identifying the correlates of fear of COVID-19 may guide future interventions to help individuals who have higher levels of fear of COVID-19.

With regard to our expectations, it may be worth noting that reporting fear of COVID-19 is generally very plausible since there was a high case fatality rate (10) (particularly among individuals with chronic conditions—which is presumably often the case among homeless individuals). A great fear of COVID-19 has also been shown in different recent international studies (summarized in a systematic review and meta-analysis) in general populations (11). Despite the fact that homeless individuals frequently suffer from challenging hygienic conditions and several chronic conditions (12), the only available previous study (7) identified a low level of fear of COVID-19 among homeless individuals. We, therefore, assume that the fear of COVID-19 is even lower due to habituation effects in our current study. This means that homeless individuals may adapt to the conditions of the pandemic.

## Materials and methods

### Sample

We used cross-sectional data from the “national survey on psychiatric and somatic health of homeless individuals during the COVID-19 pandemic” (NAPSHI). In the NAPSHI study, homeless individuals were interviewed (using a standardized questionnaire) in lodging houses, women's shelters, night shelters, drug counseling centers, and medical practices. Data collection took place in several large German cities (Hamburg, Leipzig, Frankfurt am Main, Munich, Mainz, and Augsburg) from late July to mid-September 2021. Individuals received an incentive of 5 € per half an hour. Main inclusion criteria were: aged 18 years and over and not having a permanent residence for 7 or more days. Key exclusion criteria were cognitive impairment and a current pregnancy. Items were translated (English, Bulgarian, Polish, and Russian) on site, if required.

All participants provided written informed consent prior to participation. The Ethics Committee of the Hamburg Chamber of Physicians approved this study (No.: PV7333).

### Dependent variables

To measure fear of COVID-19, participants were asked how concerned they were about contracting COVID-19 one day [four options: 1 = Not at all; 2 = a little; 3 = somewhat; 4 = severely?]. Another study also used such an assessment (7). Previous studies also used a similar item to assess fear of specific diseases (13, 14).

### Independent variables

In regression analysis, we included these independent variables: sex (men; women), age group (18–29 years; 30–49 years; 50–64 years; 65 years and over), marital status (married, living together; married, living alone; single; widowed; divorced), highest educational degree (no degree; school education; vocational training; University or University of Applied Sciences), having children (no; yes), duration of homelessness (0–11; 12–35; 36 months and above), born in Germany (no; yes), and having health insurance (no; yes).

In addition, we included factors referring to COVID-19: Vaccination against COVID-19 (no; yes), perceived own risk of contracting the coronavirus one day (single item from 1 = very low to 5 = high), the perception that being infected with the coronavirus is preventable (single item from 1 = does not apply to 7 = fully applies) and the agreement that a diagnosis of the coronavirus would ruin his/her life (single item from 1 = does not apply to 7 = fully applies). Moreover, informed by the chronic conditions included in the Survey of Health, Aging and Retirement in Europe (15), these chronic conditions were included: heart attack, high blood pressure or hypertension, high blood cholesterol, stroke or cerebral vascular disease, diabetes or high blood sugar, chronic lung disease, cancer or malignant tumor, Alzheimer's disease/dementia/memory impairment, affective or emotional disorders, liver cirrhosis/liver damage, HIV/Aids, and tuberculosis. To this end, individuals were asked to indicate which chronic conditions they had been officially diagnosed with by their doctor. Based on these chronic conditions, we created a count score.

### Statistical analysis

Sample characteristics are displayed for our analytical sample. Thereafter, the correlates of fear of COVID-19 were identified using multiple linear regressions. Some missing data occurred (mainly due to language restrictions). For example, the highest proportion of missing values occurred in the variable referring to the “agreement that a diagnosis of the coronavirus would ruin his/her life” (8.7%). In the outcome measure, the proportion of missing values equaled 6.6%. Thus, a full information maximum likelihood (FIML) approach was used to tackle missing data in a robustness check (16, 17). Additionally, ordered probit regressions were conducted in another robustness check. The significance level was set at 0.05 and Stata 16.1 (Stata Corp., College Station, Texas) was used to perform the analyses.

## Results

### Sample characteristics

In Table 1, the sample characteristics (n = 666) are given. In our analytical sample, mean age equaled 43.3 years (*SD*: 12.1 years), ranging from 18 to 80 years. Overall, 81.9% of the individuals were male.

**Table 1:** Sample characteristics.

**Variables**	**Mean** **(*SD*)/*n* (%)**
Fear of COVID-19	1.5 (0.9)
**Sex**	
Men	533 (81.9%)
Women	118 (18.1%)
Age	43.4 (12.1)
**Age group**	
18–29 years	86 (13.6%)
30–49 years	335 (53.1%)
50–64 years	181 (28.7%)
65 years and over	29 (4.6%)
**Marital status**	
Married, living together	27 (4.3%)
Married, living alone	48 (7.6%)
Single	421 (67.0%)
Widowed	16 (2.5%)
Divorced	116 (18.5%)
**Highest educational degree**	
No degree	113 (18.1%)
School education	287 (46.0%)
Vocational training	179 (28.7%)
University or University of Applied Sciences	45 (7.2%)
**Duration of homelessness**	
0–11 months	238 (38.5%)
12–35 months	157 (25.4%)
36 months and above	223 (36.1%)
**Presence of children**	
No	334 (52.3%)
Yes	305 (47.7%)
**Born in Germany**	
No	309 (48.9%)
Yes	323 (51.1%)
**Having health insurance**	
No	199 (31.4%)
Yes	435 (68.6%)
**Already vaccinated against COVID-19**	
No	257 (41.2%)
Yes	367 (58.8%)
Perceived own risk of getting the coronavirus one day (from 1 = very low to 5 = high)	1.8 (1.1)
Perception that being infected with the coronavirus is preventable (from 1 = does not apply to 7 = fully applies)	3.6 (2.5)
Agreement that a diagnosis of the coronavirus would ruin his/her life (from 1 = does not apply to 7 = fully applies)	2.3 (2.0)
Chronic conditions (count score)	1.0 (1.3)

In total, 70.9% of the homeless individuals reported no fear of COVID-19. Furthermore, 14.0% reported a little fear of COVID-19, 8.4% reported some fear of COVID-19 and 6.7% reported severe fear of COVID-19. This results in an average fear of COVID-19 score of 1.5 (*SD*: 0.9). Additional details are given in Table 1.

### Regression analysis

In Table 2, the results of multiple linear regressions are shown (with fear of COVID-19 as dependent variable). *R*^2^ was 0.32. Our linear regressions showed that a higher age group (i.e., individuals aged 50–64 years compared to individuals aged 18–29 years) reported a higher fear of COVID-19 (*β* = 0.28, *p* < 0.05). Moreover, individuals with a higher perceived own risk of contracting the coronavirus one day (*β* = 0.28, *p* < 0.001) and individuals with a higher agreement that a diagnosis of the coronavirus would ruin his/her life reported a higher fear of COVID-19 (*β* = 0.15, *p* < 0.001). The regressions with FIML to address missing (last column in Table 2) were nearly identical in terms of effect sizes and significance when compared with our linear regressions with listwise deletion to address missing data.

**Table 2:** Determinants of Fear of COVID-19.

**Independent variables**	**Fear of** **COVID-19** **(with listwise** **deletion to** **address** **missings)**	**Fear of** **COVID-19** **(with FIML to** **address** **missings)**
Sex: Women (Ref.: Men)	0.02	0.03
	(0.10)	(0.09)
Age group: −30 to 49 years (Ref.: 18 to 29 years)	0.10	0.07
	(0.10)	(0.09)
−50 to 64 years	0.28^*^	0.25^*^
	(0.12)	(0.10)
−65 years and over	0.44+	0.29
	(0.25)	(0.20)
Marital status: - Married, living alone (Ref.: Married, living together)	−0.31	0.09
	(0.25)	(0.18)
- Single	−0.17	−0.10
	(0.22)	(0.13)
- Widowed	−0.22	−0.01
	(0.29)	(0.09)
- Divorced	−0.14	0.10
	(0.23)	(0.20)
Highest educational degree: - School education (Ref.: No degree)	−0.12	−0.16+
	(0.10)	(0.09)
- Vocational training	−0.06	−0.09
	(0.12)	(0.10)
- University or University of Applied Sciences	−0.04	−0.07
	(0.19)	(0.16)
Presence of children: Yes (Ref.: No)	−0.05	−0.09
	(0.08)	(0.07)
Duration of homelessness: - 12 to 35 months (Ref.: 0–11 months)	0.10	0.04
	(0.09)	(0.08)
− 36 months and above	0.15+	0.12
	(0.09)	(0.08)
Born in Germany: Yes (Ref.: No)	0.01	0.07
	(0.09)	(0.08)
Having health insurance: Yes (Ref.: No)	−0.02	−0.03
	(0.09)	(0.08)
Already vaccinated against COVID-19: Yes (Ref.: No)	−0.13+	−0.10+
	(0.07)	(0.06)
Perceived own risk of getting the coronavirus 1 day (from 1 = very low to 5 = high)	0.28^***^	0.31^***^
	(0.04)	(0.04)
Perception that being infected with the coronavirus is preventable (from 1 = does not apply to 7 = fully applies)	0.03	0.02
	(0.02)	(0.01)
Agreement that a diagnosis of the coronavirus would ruin his/her life (from 1 = does not apply to 7 = fully applies)	0.15^***^	0.13^***^
	(0.03)	(0.02)
Chronic conditions (count score)	0.01	0.01
	(0.03)	(0.03)
Constant	0.87^**^	0.74^**^
	(0.32)	(0.25)
Observations	488	666
*R* ^2^	0.32	0.32

Findings of Multiple Linear Regressions.

Comments: Unstandardized beta-coefficients are reported; robust standard errors in parentheses;

*** p < 0.001,

** p < 0.01,

* p < 0.05,

+ p < 0.10.

The findings of the ordered probit regressions were virtually the same compared to the multiple linear regressions mentioned before in terms of significance (results not shown, but available upon request). However, the variable referring to the perception that being infected with the coronavirus is preventable gained statistical significance. More precisely, a higher perception was associated with higher fear of COVID-19 (*p* = 0.01).

## Discussion

Based on data from the NAPSHI study, our aim was to investigate the prevalence and the correlates of fear of COVID-19 among homeless individuals in several large cities in Germany in Mid-2021. About 7 out of 10 homeless individuals reported no fear of COVID-19. Moreover, regressions showed that fear of COVID-19 was higher among individuals aged 50–64 years (compared to individuals aged 18–29 years), among individuals with a higher perceived own risk of contracting the coronavirus one day as well as among individuals with a higher agreement that a diagnosis of the coronavirus would ruin his/her life. Our current study markedly extends the very restricted knowledge regarding fear of COVID-19 among homeless individuals.

In light of the high morbidity of homeless individuals, it may sound contradictory that most individuals did not report fear of COVID-19 at all in our study. This may be explained by the underestimation of the risk of getting infected with SARS-CoV-2 among the homeless—perhaps due to mental disorders (7) or by the presumably more difficult access to information (e.g., daily newspapers, internet, or television) on the health consequences caused by the virus. The low level of fear of COVID-19 is, however, in accordance with the only existing previous study examining fear of COVID-19 among homeless individuals in Hamburg (Germany) in May 2020 (7). The fact that even more individuals did not report fear of COVID-19 may be mainly explained by the fact that they adapt to the conditions of the pandemic—a similar habituation phenomenon has been identified for fear of COVID-19 among the general adult population in Germany (18).

In our current study, the fear of COVID-19 was higher among individuals aged 50–64 years (compared to individuals aged 18–29 years). Such an association may be explained by the fact that older individuals have a more realistic perception of the potential risk of an infection, whereas homeless individuals aged 18–29 years may neglect such risks associated with an infection. However, studies based on data from the general adult population mostly found an association between younger samples and higher fear of COVID-19 [e.g., (19)]. Thus, future research is required in this area.

Former research revealed a link between perceived risk of an illness and fear of other illnesses such as dementia (20). The previous German study among homeless individuals also found such an association between perceived risk of contracting COVID-19 one day and higher fear of COVID-19 (7). Thus, our findings regarding an association between higher perceived own risk of contracting the coronavirus one day and higher fear of COVID-19 are quite plausible to us. Likewise, prior research underlined the relevance of consequences of chronic conditions for fear of illnesses such as cancer (21) or COVID-19 (7). Thus, our present findings are in line with former research and appear to be plausible.

Several strengths and limitations of our study should be highlighted. With regard to strengths, it should be stressed that data (with a quite large sample size) were taken from homeless individuals—which are commonly difficult to reach. Furthermore, data were collected during the pandemic (over nearly 2 months with steadily relatively low incidence rates). A rather high response rate was identified. As one of the very few studies, the prevalence and correlates of fear of COVID-19 in such a vulnerable group were quantified. Several correlates were included in our regression model. Due to the presence of some missing data, a FIML approach was used to address missing data (16, 17). With regard to limitations, in accordance with recent studies [such as (22)] and comparable to previous research examining fear of specific illnesses (13, 14, 20), a single-item with high face validity was used to quantify fear of COVID-19. Such an assessment was favored over more complex tools in this particularly vulnerable target group to avoid non-response and to reduce the risk of misinterpretations. Nevertheless, it should be acknowledged that other tools exist such as the Coronavirus Anxiety Scale (23). Restrictions exist regarding causality due to the cross-sectional design. Additionally, we used a quantitative approach with its known limitations (e.g., it may be difficult to understand fear of COVID-19 among homeless individuals). Thus, upcoming qualitative studies are required to get a better understanding of fear of COVID-19 among homeless individuals.

## Conclusions

Only a small proportion of homeless individuals reported fear of COVID-19 in Germany in mid-2021. Policymakers and social services should be also aware of the factors associated with (higher levels of) fear of COVID-19 among homeless individuals in Germany. Such knowledge about the correlates of higher levels of fear of COVID-19 may be helpful for addressing certain risk groups (e.g., homeless individuals aged 50–64 years). In a further step, avoiding extraordinarily high levels of fear of COVID-19 may be beneficial to avoid irrational thinking and acting regarding COVID-19 in this group.

## Data availability statement

The datasets presented in this article are not readily available because the datasets analyzed during the current study are not publicly available due to ethical restrictions involving patient data but are available from the corresponding author on reasonable request. Requests to access the datasets should be directed to AH, a.hajek@uke.de.

## Ethics statement

The studies involving human participants were reviewed and approved by the Ethics Committee of the Hamburg Chamber of Physicians. The patients/participants provided their written informed consent to participate in this study.

## Author contributions

KP, FB, FH, AH, and H-HK: conceptualization. AH: methodology and formal analysis and writing—original draft preparation. AH, FB, VR, KD, WG, AB, VK, KP, CS, BO, FH, and H-HK: writing—review and editing. H-HK: supervision. FH, FB, and KP: funding acquisition. All authors have read and agreed to the published version of the manuscript.

## Funding

This study was financially supported by the Volkswagen Foundation. The funders had no influence on study design, data collection, analysis, decision to publish, or preparation of the manuscript.

## Conflict of interest

The authors declare that the research was conducted in the absence of any commercial or financial relationships that could be construed as a potential conflict of interest.

## Publisher's note

All claims expressed in this article are solely those of the authors and do not necessarily represent those of their affiliated organizations, or those of the publisher, the editors and the reviewers. Any product that may be evaluated in this article, or claim that may be made by its manufacturer, is not guaranteed or endorsed by the publisher.
